# Effect of a combination of pea protein, grape seed extract and lactic acid in an in vivo model of bacterial vaginosis

**DOI:** 10.1038/s41598-023-28957-7

**Published:** 2023-02-17

**Authors:** Marika Lanza, Sarah Adriana Scuderi, Anna Paola Capra, Giovanna Casili, Alessia Filippone, Michela Campolo, Salvatore Cuzzocrea, Emanuela Esposito, Irene Paterniti

**Affiliations:** Department of Chemical, Biological, Pharmaceutical and Environmental Sciences, University of Messina, Viale Ferdinando Stagno D ’Alcontres, 31, 98166 Messina, Italy

**Keywords:** Drug discovery, Microbiology

## Abstract

Bacterial vaginosis (BV) is a common vaginal dysbiosis characterized by a malodorous discharge and irritation. The imbalance of the vaginal microbiota plays a key role in the development of BV**.** It has been demonstrated that *Gardnerella vaginalis* (GV), a facultative anaerobic bacillus, is involved in BV. Due to the rising number of antimicrobial-resistant species, recurrence of BV is becoming more frequent in women; thus, alternative treatments to antibiotics are needed. Natural substances have recently shown a great efficacy for the treatment of vaginal dysbiosis. Thus, this study aimed to investigate the beneficial effect of a product containing pea protein (PP), grape seed extract (GS) and lactic acid (LA) in an in vivo model of Gardnerella vaginalis-induced vaginosis by intravaginal administration of GV suspension (1 × 10^6^ CFU/20 µL saline). Our results demonstrated that the product containing PP, GS and LA significantly reduced GV proliferation. More specifically, it significantly preserved tissue architecture and reduced neutrophil infiltration, inflammatory markers and sialidase activity when used both as a pre- or a post-treatment. Moreover, the product displayed strong bioadhesive properties. Therefore, our data suggested that the product containing PP, GS and LA could be used as alternative preventive or curative treatment for the management of BV.

## Introduction

Bacterial vaginosis (BV) is the most common vaginal dysbiosis in women of childbearing age^1^, and it is estimated to occur in 5–70% of women^1,2^. BV is caused by an imbalance of the vaginal microbiota^2^ and is characterized by a thin, gray/off-white, malodorous adherent vaginal discharge^3,4^. The abnormal vaginal discharge results in part from the degradation of the protective vaginal mucin gel, induced by mucin-degrading enzymes produced by BV-associated bacteria^5^. BV has been associated with urinary tract infections, higher risk of sexually transmitted infections, infertility, preterm birth, intrauterine and intraamniotic infections, as well as cervical infections, dysplasia, and cancer^6–9^. It has been demonstrated that vaginal microbiota of healthy women is composed mainly of lactobacilli which maintain the acidic milieu of the vagina through lactic acid production inhibiting the growth of harmful pathogens^10^. Alteration of the vaginal microbiota causes change in pH, which allows a variety of anaerobes and facultative bacteria to overgrow and cause chronic infection as well as abnormal vaginal discharge^11,12^. Bacteria that colonize vaginal mucosa are mainly Gram-positive cocci or anaerobic species such *as Prevotella*^13^*, Mobiluncus*^14^*,* and the facultative anaerobic bacillus *Gardnerella vaginalis* (GV)^15^. Particularly, GV has been extensively studied because it has been found from the vaginal samples of almost all women with BV^16–18^. Over the past decades, several classifications have been developed to delineate subgroups or “clades” of GV^19^. In 2019, Vaneecoutte et al.^20^ identified three other BV-associated *Gardnerella* species based on whole genome sequence comparison, *G. leopoldii, G. piotii,* and *G. swidsinskii*. Plummer and colleagues^21^ revealed that *G. leopoldii,* and *G. swidsinskii* were not strongly associated with BV or the absence of Lactobacillus, supporting the existence of symbiotic and pathogenic subtypes of *Gardnerella* spp.^22^. Nevertheless, a recent study conducted by Hill et al.^23^ demonstrated that a higher abundance of *G. swidsinskii* is associated to several BV symptoms, including abnormal and malodourous vaginal discharge. BV is characterized by a polymicrobial biofilm, a community of microorganisms primarily constituted by GV clusters, strongly adhered to the vaginal epithelium^24^. The interactions between bacteria in multi-species biofilms contribute to enhance antimicrobial tolerance as standard antibiotics are unable to completely eradicate the vaginal biofilm, leading to relapses^24^. As biofilms allow *Gardnerella* spp. to persist in recurrent BV, it is important to identify new treatments capable to counteract BV-biofilm^24,25^. Currently, the therapeutic agents available for the management of BV are topical agents containing lactic acid, clindamycin and/or orally administered antibiotics^26^, among others. However, oral administration of clindamycin (CLIN) and/or metronidazole (MET) long-term could negatively impact the gut microbiome and increase BV recurrence^27^, due to the rising number of antimicrobial resistant species^28^; therefore, alternative treatments are needed for a better management of BV^26,29^. In the last decade, more attention was given to the use of natural substances for the treatment of vaginal dysbiosis, including BV^25,30–32^. Pea protein (PP) is derived from the plant of *Pisum sativum*; it is rich in fiber and represents an important source of essential amino acids such as lysine^33^. PP has shown to exert several health benefits including protective action on mucosal tissues^34^. Grape seed extract (GS) has gained great interest since it is rich in antioxidants and oligomeric proanthocyanidins^34^, while lactic acid (LA) is well-known for its ability to maintain the physiological vaginal pH and regulate the vaginal immune response^10^. Based on these findings, the aim of this study was to evaluate the efficacy of a product containing PP, GS and LA in an in vivo model of GV-induced bacterial vaginosis.

## Results

### Effect of PP, GS and LA on GV adhesion on vaginal tissue

**Figure 1:**
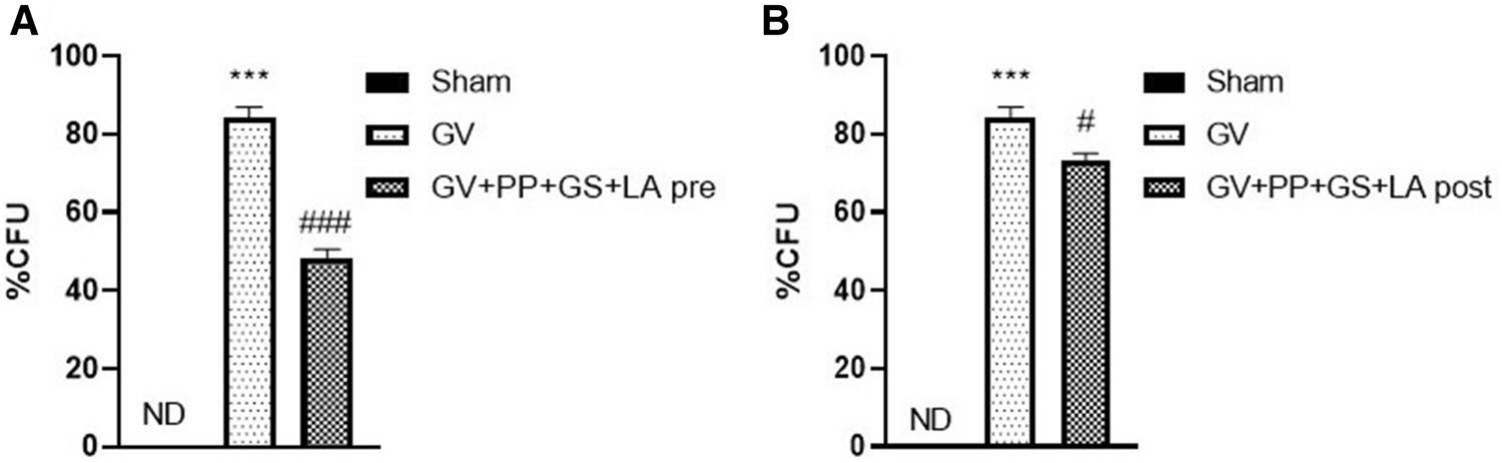
Effect of the product containing PP, GS, and LA on % CFU evaluation. The treatment with the product containing PP, GS and LA was able to reduce GV growth in a pre-treatment condition (**A**) and also in post-treatment condition (**B**). (**A**) ***p < 0.001 vs Sham; ###*p* < 0.001 vs GV group; (**B**) ***p < 0.001 vs Sham; #*p* < 0.05 vs GV group.

At the end of experiment, serial dilutions of vaginal washes onto NYC-III agar plates containing 1 mg/mL streptomycin and 4 mg/L amphotericin (both from Sigma- Aldrich) were plated to evaluate GV counts. Both the pre- and post-treatment with PP, GS and LA were significantly effective in reducing bacterial counts compared to the GV group. Notably, the pre-treatment was more effective in treating the infection reaching a reduction of the bacterial count by 35.85% compared to the post-treatment group which showed a reduction of 11%. (Fig. 1A, B).

### Effect of PP, GS and LA on histological damage induced by GV infection

**Figure 2:**
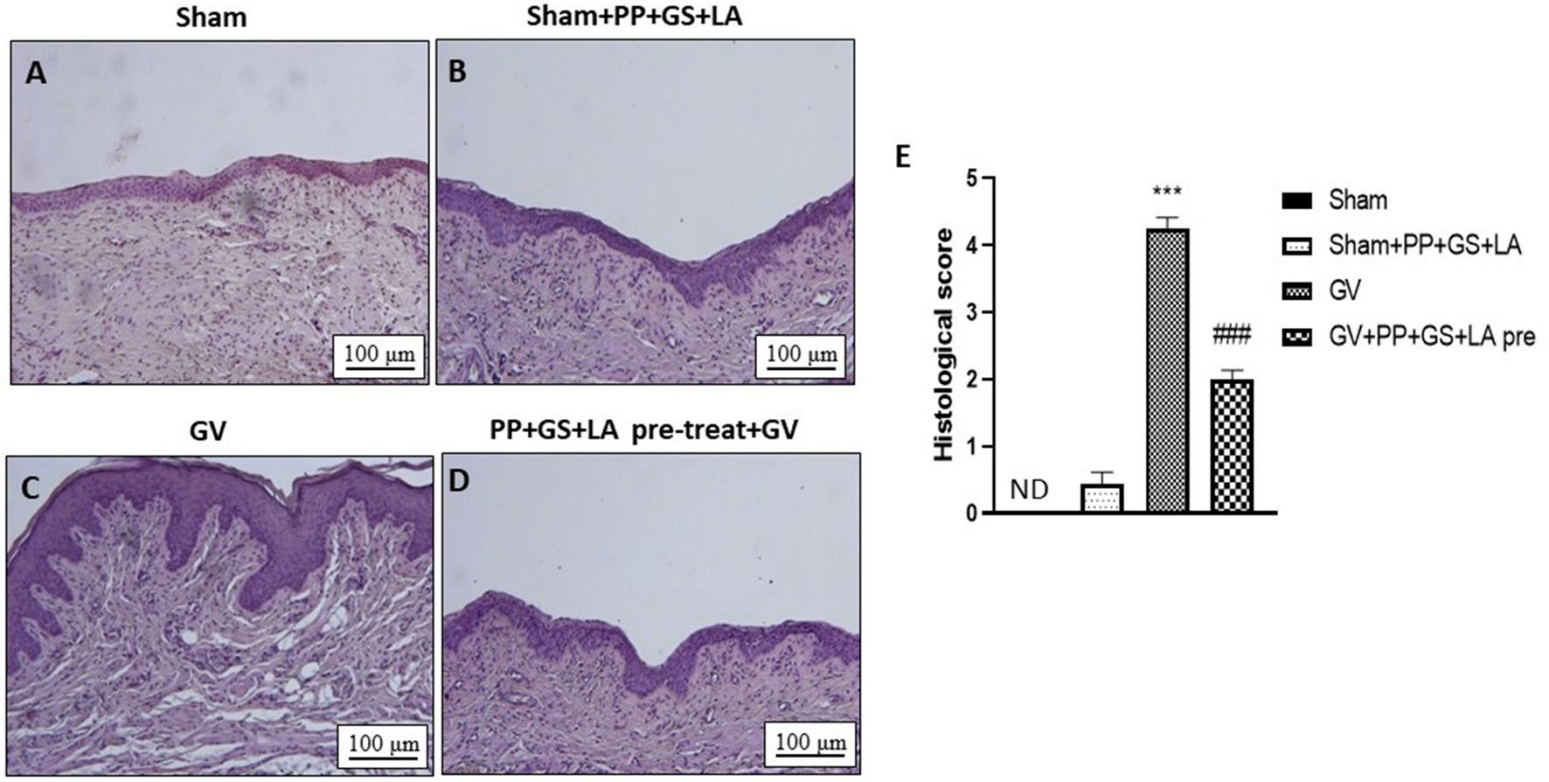
Effect of the product containing PP, GS, and LA on histological damage in pre-treatment condition. The treatment with the product containing PP, GS and LA (**D**) given 1 h before GV infection was able to significantly preserve tissue architecture compared to GV group (**C**). No significance difference was observed in the sham group treated with PP, GS and LA group (**B**) compared to sham group alone (**A**). (**E**) ***p < 0.001 vs Sham; ###*p* < 0.001 vs GV group.

**Figure 3:**
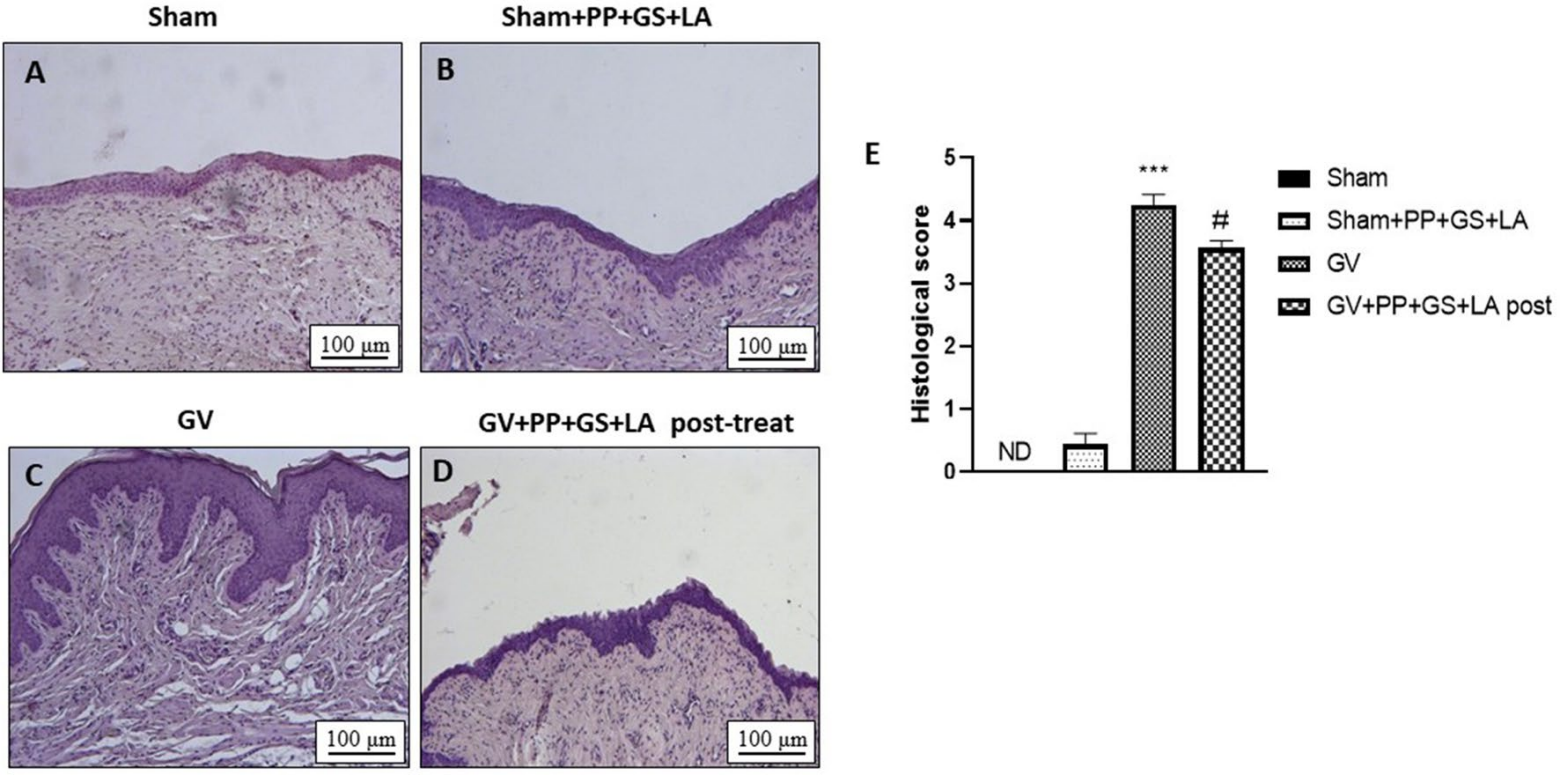
Effect of the product containing PP, GS, and LA on histological damage in post-treatment condition. The treatment with the product containing PP, GS and LA (**D**) was able to reduce histological damage post GV infection (**C**). No significance difference was observed in the sham group treated with PP, GS and LA (**B**) compared to sham group alone (**A**). (**E**) ***p < 0.001 vs Sham; #*p* < 0.05 vs GV group.

Histological examination of vaginal tissues revealed characteristic pathological changes 7 days after infection (Figs. 2C, 3C), compared to sham group (Figs. 2A, 3A). The product containing PP, GS and LA was able to significantly preserve tissue architecture both as a pre-treatment (Fig. 2D) and post-treatment (Fig. 3D). No significance difference was observed in the sham groups treated with PP, GS and LA (Figs. 2B, 3B). However, the decrease in the histological score was more evident in the pre-treatment group (Fig. 2E) compared to post-treatment group (Fig. 3E).

### Effect of PP, GS and LA on myeloperoxidase activity of GV infection

**Figure 4:**
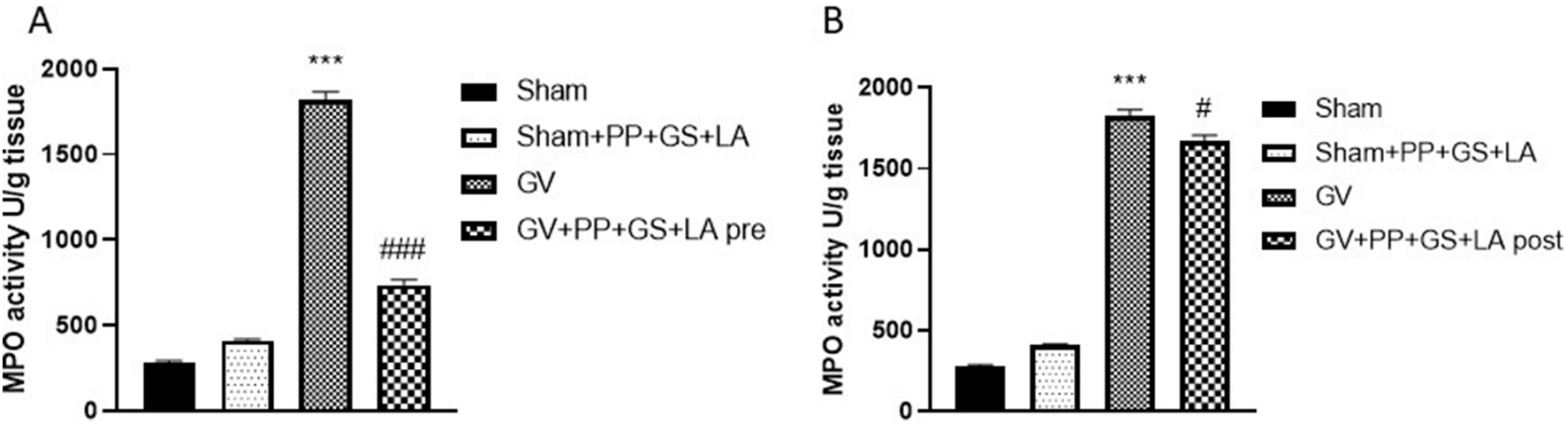
Effect of the product containing PP, GS and LA on MPO activity. The product containing PP, GS and LA significantly decreased MPO activity in pre-treatment condition compared to GV group (**A**). Moreover, the product containing PP, GS and LA was able to reduce MPO activity post GV infection (**B**). (**A**) ***p < 0.001 vs Sham; ###*p* < 0.001 vs GV group. (**B**)***p < 0.001 vs Sham; #*p* < 0.05 vs GV group.

MPO activity, an index of polymorfonucleate infiltration associated to inflammation, was evaluated in vaginal tissue homogenates from GV-infected mice. Our results demonstrated that the GV-infected mice group was characterized by an elevated MPO activity at 7 days post-infection. However, the product containing PP, GS and LA was able to significantly decrease MPO activity in pre-treatment condition as shown in the Fig. 4A. Additionally, our data demonstrated that the product containing PP, GS and LA was able to reduce MPO activity post GV infection, as shown in the Fig. 4B.

### Effect of PP, GS, and LA on sialidase activity of GV infection

**Figure 5:**
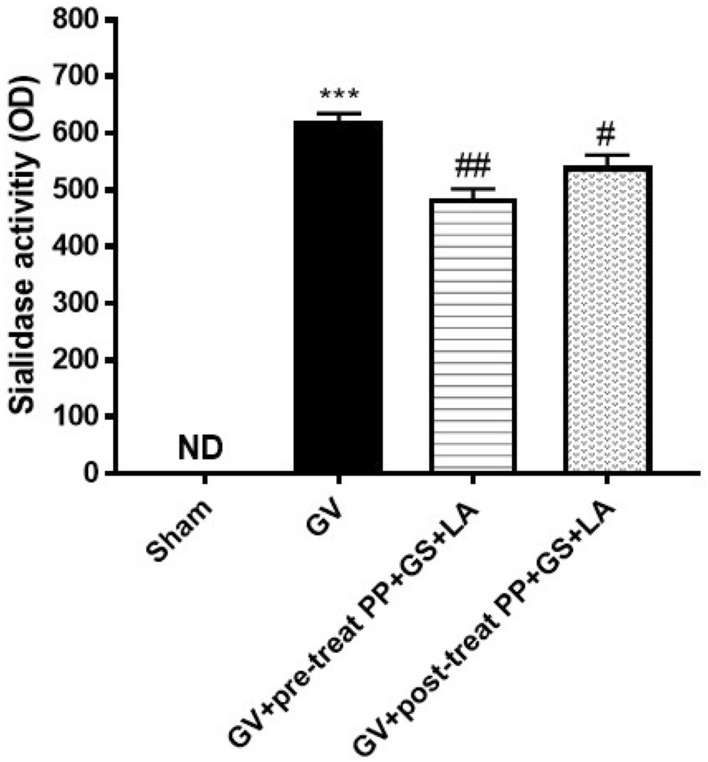
Effect of the product containing PP, GS and LA on sialidase activity. The product containing PP, GS and LA significantly decreased sialidase enzyme activity compared to GV infected mice group in pre-treatment and post-treatment conditions. ***p < 0.001 vs Sham; #*p* < 0.05 vs GV group; ##*p* < 0.01 vs GV group.

Sialidase activity is an important hallmark feature of bacterial vaginosis^35^ and was evaluated using vaginal washes from mice. The obtained results showed that the pre-treatment with the product containing PP, GS and LA was able to significantly inhibit sialidase enzyme activity compared to GV infected mice group as shown in the Fig. 5. Moreover, we evaluated the effect of the product containing PP, GS and LA on sialidase activity also after GV infection, showing that PP, GS and LA significantly decreased sialidase enzyme activity compared to GV infected mice group (Fig. 5).

### Effect of PP, GS and LA on mucoadhesion of GV infection

**Figure 6:**
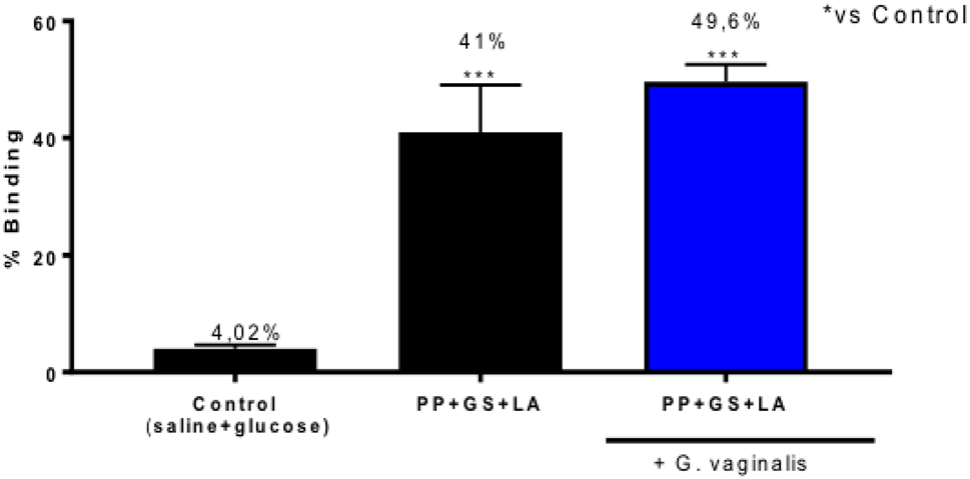
Mucoadhesive effect of the product containing PP, GS, and LA. The treatment with the product containing PP, GS and LA showed a significant binding percentage, respectively to respectively to 41% in absence of *G. vaginalis* infection and 49.6% in presence of *G. vaginalis* infection compared to control group (saline + glucose). ***p < 0.001 vs control group.

The everted vaginal sac assay evaluated the bioadhesive interaction of the cream with everted vaginal tissue in infected and non-infected mice. The results were presented as percent binding, which was derived from subtracting the weight of the washed and lyophilized cream particles remaining in the tube after incubation from the original tare weight of the cream particles. A high percentage of binding indicated strong bioadhesion of the particles to mucosal tissue. The treatment with a product containing PP, GS and LA showed a significant binding percentage, respectively to 41% in absence of G. vaginalis infection and 49.6% in presence of G. vaginalis infection compared to control group (saline + glucose), demonstrating a strong bioadhesion to vaginal mucosal tissue^36^ (Fig. 6).

## Discussion

BV is caused by an overgrowth of some bacteria naturally found in the vaginal microbiota^3,37^. The vaginal mucosa represents an important and complex ecosystem dominated by *Lactobacillus* strains and containing a small number of fungi and balanced microbial communities which are vital for the health of the female reproductive tract^38^. However, even though 30% of women possess a healthy vaginal microbiota, a lack of significant concentrations of *Lactobacillus* species has been found compared to other facultative or strictly anaerobic bacteria^39,40^. The vaginal mucosal barrier is a multifaceted system comprising of a mucus layer and an epithelium layer. The latter guarantees mechanical protection against harmful pathogens^41^ as it embodies antimicrobial peptides (AMPs) associated with the innate and the adaptive immune system which represent the first line of defence against pathogen invasion^42^.The standard treatment for BV includes the use of antibiotics such as: metronidazole (MET), clindamycin (CLIN) or alternatively tinidazole (TI), which can be administered orally or intravaginally^43^. A systematic review conducted by Oduyebo et al.^44^ revealed that CLIN and MET, either in oral or vaginal formulations, were equally effective in treating BV, and that CLIN caused a lower rate of adverse events than MET. Studies suggest a lack of efficacy of traditional antimicrobial treatments and high recurrence rates of BV, of 30% and 76% within 4 weeks and 6 months, respectively, due to their inability to completely eradicate the vaginal biofilm. In addition, the negative effect of antibiotics on healthy vaginal microbiota impact therapeutical efficacy and symptom relief even further^43,45^. This ecosystem can be altered by several factors, including alterations of CO_2_/O_2_ levels and pH in the vaginal lumen. Considering an imbalance of vaginal mucosal barrier plays a pivotal role in BV pathogenesis^10^, novel treatments strengthening the vaginal barrier could represent an alternative solution to avoid and/or reduce the onset and progression of GV infections. Thus, there is a great need to source and develop new compounds to combat them^46,47^. Traditionally, natural compounds have been used for the treatment of vaginal dysbiosis^43^. These include plant extracts, such as *Prangos ferulacea, Myrtus communis*, *Zataria multiflora*, essential oils, small antimicrobial peptides of animal origin, bacteriocins and various groups of plant compounds (such as triterpenoids, phenols, and flavonoids) with antimicrobial and antiviral activity^46,48^. In particular, studies conducted by Machado and Rosca^25,32^ demonstrated the potential use of Thymbra capitata essential oil (EO) as a novel therapeutic agent in the treatment of BV thanks to its antimicrobial effect against several bacterial species associated with BV including *Gardnerella* species. The natural products can be used in combination with MET or probiotics to improve the efficacy of antimicrobial therapy and reduce the recurrence of vaginal infections^43^. Natural compounds are preferred over synthetic drugs to avoid any contraindications or side effects related to the intake of synthetic substances, which makes them attractive to patients^46^. In this context, recent reports have highlighted the health-promoting properties of some natural compounds in the management of BV^25,34^. A previous study performed by Esposito et al.^34^ demonstrated the beneficial effects of grape seed extract and pea protein in a murine model of vulvovaginal candidiasis, showing the ability of these substances to create a protective barrier on vaginal mucosa against fungal infection. Furthermore, LA has also gained considerable interest in the field of medicine due to its ability to maintain an acidic environment (pH 3.5–4.5) in the vagina to restrict the growth of fungi such as *C. glabrata* and *C. albicans*^47,49^. In particular, vaginal gels containing LA have an optimal adherence to the mucosa and enhance the vaginal defence mechanisms^50^. In addition, the optimal safety profile of PP, GS, and LA was demonstrated^34,47,51^ suggesting their possible use as a therapeutic alternative for the management of vaginal dysbiosis including BV. Based on these findings, the efficacy to manage BV-associated symptoms utilizing the aforementioned substances were investigated. Firstly, we evaluated GV proliferation through CFU counts, demonstrating that the product containing PP, GS and LA was able to significantly reduce vaginal bacterial growth in both pre-treatment and post-treatment conditions due to its ability to form a protective mucomimetic barrier on the vaginal mucosa. Furthermore, it has been demonstrated that BV causes alternations to the vaginal tissue^35^, characterized by the presence of edema, epithelial thickness and polymorphonuclear (PMN) cell infiltration^35^. In this context, our results demonstrated that the pre-treatment with the product containing PP, GS and LA was able to preserve vaginal tissue architecture, reducing epithelium thickness, compared to the GV infection group. Moreover, PP, GS and LA exerted a protective effect also in post-treatment condition. The development of histological damage after GV infection was accompanied by an increase of MPO activity. Here the treatment with PP, GS and LA demonstrated to be effective in reducing MPO activity, with a marked decrease in the pre-treatment group compared to the post-treatment group.

A relevant hallmark feature of BV is the presence of high levels of sialidase activity in vaginal fluid compared to specimens from women with normal microbiota^35^. Production of sialidase enzyme by isolated BV-associated bacteria grown in culture strongly suggests that BV-associated sialidases are bacterial in origin^52^. Bacterial sialidases have been characterized as virulence factors in bacterial infections of various mucosal sites^52,53^. Here, PP, GS and LA significantly inhibit sialidase activity, particularly at 1 h before GV infection. Additionally, the bioadhesive properties of the product containing PP, GS and LA with vaginal mucosa were evaluated. Mucosal membranes of human organism, including vaginal tissues, are characterized by an epithelial layer whose surface is covered by mucus^36,38^. The mucus contains glycoproteins, lipids, inorganic salts and 95% water by mass, which makes it a highly hydrated system and relatively permeable^36^. Our data demonstrated that the product containing PP, GS and LA possesses a strong bioadhesive potential to vaginal mucosal tissue, exerting a muco-protective barrier function.

Although many natural products have been identified for the treatment of BV, some of them, such as probiotics, have not been shown to be effective when administered alone^29^. It has been demonstrated that a single strain or mixture of *Lactobacilli* exerts many benefits in BV patients through lactic acid production; however, probiotics only contain bacterial strains without other potential beneficial factors, which poses a limitation for BV management^29^. Notably, our study demonstrates the beneficial effects of PP, GS, and LA, which provide a barrier-forming mechanism of action and can be considered an alternative therapeutic strategy for the treatment of BV to re-establish the pH and integrity of the vaginal mucosa. Preclinical models come with a set of limitations lacking translational reproduction of human diseases. Nonetheless, the association of PP, GS and LA could pave the way to better understanding new strategies of modulating bacterial infections, thus reducing the frequency and severity of symptoms in BV patients. It would be intriguing to further understand how PP, GS and LA could restore the balance of the vaginal microbiota by assessing resident microbial communities. Understanding the effects of PP, GS and LA in other bacteria-associated vaginal dysbiosis, such as *Prevotella* and *Mobiluncus* species or *Chlamydia trachomatis* and *Neisseria gonorrhoea* infections^54^ may also provide a deeper understanding on ways to maintain vaginal health, reduce the risk of relapse and improve the quality of life in BV patients. In conclusion**, t**he obtained data demonstrated that the pre-treatment with a product containing PP, GS, and LA could decrease the engraftment of GV; while if used as a post treatment, it represents an impediment for the colonization of GV in the vaginal environment. Moreover, our results suggest that the product is able to preserve vaginal tissue architecture and avoid invasion and bacterial growth if used as both preventive or curative treatment for the management of BV. Thus, PP, GS and LA could represent an alternative therapeutic strategy to prevent and treat BV, significantly decreasing the risk of developing antimicrobial resistance that may come with conventional therapies.

## Material and methods

### Animals

Seven-week-old female C57BL/6 mice weighing 19–22 g were housed in wire cages under climate-controlled conditions (at 50 ± 10% humidity and 20–22 °C) in accordance with ARRIVE guidelines, fed with standard laboratory chow, and water ad libitum. The experimental protocol was approved by the Ethics Committee of University of Messina (n°409/2022-PR). The Animal experiments followed Italian regulations on protection of animals used for experimental and other scientific purposes (DM 116192) as well as EU regulations (OJ of EC L 358/1 12/18/1986). The animals used for this study were randomly selected from those suitable and available at that time.

### Gardnerella vaginalis growth condition

*Gardnerella vaginalis* (GV) KCTC5096 was obtained from the Korean Collection for Type Cultures (KCTC, Daejeon, Korea). The GV strain was cultured in a modified brain–heart infusion (mBHI; Difco, Detroit, MI, USA) broth containing 10% (v/v) horse serum (Life Technologies Co., Grand Island, NY, USA), 1% (w/v) yeast extract (Difco, Detroit, MI, USA), 0.1% (w/v) maltose, and 0.1% (w/v) glucose, and cultivated at 37 °C for 48 h under anaerobic conditions (BD GasPakTM EZ pouch systems, BS, USA)^55^.

### Gardnerella vaginalis-induced vaginosis model

Mice were treated subcutaneously with β-Estradiol-3-benzoate (0.5 mg/0.1 mL) 72 h before GV infection, and then a suspension of GV (1 × 10^6^ CFU /20 µL saline) was administered intravaginally^35,56^. The control groups were treated with saline instead of the GV suspension. The product containing PP, GS and LA was administered intravaginally once a day for 7 consecutive days 1 h before GV-infection at the dose of 0.2 g/mouse. For another set of experiments, the product containing PP, GS and LA was administered once a day for 7 consecutive days after infection at the same dose. GV-infected control group was treated with saline (vehicle). Mice were sacrificed as previously described by Valentim et al.^57^ 24 h after the final test item treatment. After placing the animal in the sevoflurane chamber to induce unconsciousness, cervical dislocation was performed^57^. GV was chosen to perform the BV model because it is considered one of the most common bacteria involved in BV^17,18^.

### Experimental groups

Mice were divided into 5 experimental groups:

- group 1: mice received vehicle (saline) without GV infection for 7 days (n = 5)
- group 2: mice received PP, GS and LA without GV infection for 7 days (n = 5)
- group 3: mice received PP, GS and LA 1 h before GV infection for 7 days (pre-treatment) (n = 10)
- group 4: mice were infected with GV without PP, GS, and LA treatment (n = 10)
- group 5: mice received PP, GS and LA beginning the day after infection GV for 7 days (post-treatment) (n = 10).

### CFU evaluation

Serial dilutions of vaginal washes onto NYC-III agar plates + 1 mg/mL streptomycin and 4 mg/L amphotericin (both from Sigma-Aldrich) were performed to evaluate GV proliferation as previously described^58^. The plates were incubated at 37 °C under anaerobic conditions. This process was carried out with two replicates in at least three independent assays. The final concentration of bacteria-free supernatants per well is reported based on the 10% v/v dilution of the bacterial culture density (CFU/ml) and then expressed as %^59^.

### Histological evaluation

Histological evaluation was performed as previously described by Lanza et al.^60^. The vaginal tissues were fixed with 10% neutral formalin, embedded in paraffin, and sectioned at 7 µm. Sections were stained with hematoxylin–eosin (H&E). All sections were evaluated using an AxioVision microscope (Axostar Plus equipped with Axio-Cam MRc, Zeiss, GE, Germany). The histological results are shown at 10× magnification (100 μm of the Bar scale). The parameters evaluated to assess tissue architecture were: epithelial thickness, edema and polymorphonuclear (PMN) cell infiltration^35,58,61^. Infection degree was scored as (1) absent (score of 0), meaning no yeast elements; (2) mild (score of 1), meaning 1–30 yeast elements in the vaginal lumen and cornified epithelial layer, or ≥ 5 in the upper 1/3 layer of the vaginal mucosa; (3) moderate (score of 2), meaning 31–60 yeast elements in the vaginal lumen and cornified epithelial layer or ≥ 5 in the upper 2/3 layer of the vaginal mucosa; and (4) severe (score of 3), meaning > 60 yeast elements in the vaginal lumen and cornified epithelial layer or ≥ 5 in the whole layer of the vaginal mucosa^62^. The slides were analyzed by a pathologist blinded to the treatment groups.

### Myeloperoxidase (MPO) activity

MPO activity, an index of polymorphonuclear cell accumulation, was determined as previously described by Lanza et al.^60^. The rate of change in absorbance was measured spectrophotometrically at 650 nm. MPO activity was measured as the quantity of enzyme degrading 1 mM of peroxide min − 1 at 37 °C and was expressed in units per gram weight of wet tissue.

### Sialidase activity assay

The activity of sialidase, an enzyme commonly used as diagnostic marker of BV^63^, was evaluated as previously described^64^. In the presence of BV, bacterial sialidase plays an important role in biofilm formation on vaginal epithelium^63^. To quantitatively assay the vaginal wash samples for sialidase activity, 100 µL of the specimen were incubated on a microplate at room temperature and placed on a shaker with 100 µL of the substrate 2-(3′-methoxyphenyl)-*N*-acetyl-d-neuraminic acid (Sigma, St. Louis) dissolved in 0.05 mol/L sodium acetate, pH 5.0.16. After 1 h of incubation 50 µL of 4 mmol/L 4-aminoantipyrine and 50 µL of 6 mmol/L potassium ferricyanide were added. A duplicate sample lacking the substrate served as sample blank. Absorbances were read at 492 nm.

### Mucoadhesion test

Mucoadhesion test was performed as previously described by Gremião et al.^36^. The everted vaginal sac assay evaluated the bioadhesive interaction of a product containing PP, GS, and LA with everted vaginal tissue in infected and non-infected mice.

### Materials

The product containing PP, GS and LA was kindly provided by DEVINTEC SAGL (Lugano, Switzerland). All chemicals were obtained from the highest grade of commercial sources. All stock solutions were prepared in non-pyrogenic saline (0.9% NaCl; Baxter, Liverpool, UK).

### Statistical evaluation

All data were expressed as the mean ± SEM. The results were analysed with GraphPad 7 software, using one-way analysis of variance (ANOVA), followed by a Bonferroni post hoc test for multiple comparisons. A *p*-value of less than 0.05 was considered significant.

## Data Availability

The authors declare that all data and materials supporting the findings of this study are available within the article.

## References

[1] Ranjit E, Raghubanshi BR, Maskey S, Parajuli P (2018). Prevalence of bacterial vaginosis and its association with risk factors among nonpregnant women: A hospital based study. Int. J. Microbiol..

[2] Javed A, Parvaiz F, Manzoor S (2019). Bacterial vaginosis: An insight into the prevalence, alternative treatments regimen and it's associated resistance patterns. Microb. Pathog..

[3] Kairys, N. & Garg, M. Bacterial vaginosis. In *StatPearls* (Treasure Island, 2022).

[4] Engberts MK, Boon ME, van Haaften M, Heintz AP (2007). Symptomatic candidiasis: Using self sampled vaginal smears to establish the presence of *Candida*, *Lactobacilli*, and *Gardnerella vaginalis*. Diagn. Cytopathol..

[5] Olmsted SS, Meyn LA, Rohan LC, Hillier SL (2003). Glycosidase and proteinase activity of anaerobic gram-negative bacteria isolated from women with bacterial vaginosis. Sex Transm. Dis..

[6] Morrill S, Gilbert NM, Lewis AL (2020). *Gardnerella vaginalis* as a cause of bacterial vaginosis: Appraisal of the evidence from in vivo models. Front. Cell Infect. Microbiol..

[7] Sharami SH, Afrakhteh M, Shakiba M (2007). Urinary tract infections in pregnant women with bacterial vaginosis. J. Obstet. Gynaecol..

[8] Kovachev SM (2020). Cervical cancer and vaginal microbiota changes. Arch. Microbiol..

[9] Harmanli OH, Cheng GY, Nyirjesy P, Chatwani A, Gaughan JP (2000). Urinary tract infections in women with bacterial vaginosis. Obstet. Gynecol..

[10] Amabebe E, Anumba DOC (2018). The vaginal microenvironment: The physiologic role of Lactobacilli. Front. Med. (Lausanne).

[11] Valenti P, Rosa L, Capobianco D, Lepanto MS, Schiavi E, Cutone A, Paesano R, Mastromarino P (2018). Role of lactobacilli and lactoferrin in the mucosal cervicovaginal defense. Front. Immunol..

[12] van de Wijgert J (2017). The vaginal microbiome and sexually transmitted infections are interlinked: Consequences for treatment and prevention. PLoS Med..

[13] Randis TM, Ratner AJ (2019). Gardnerella and prevotella: Co-conspirators in the pathogenesis of bacterial vaginosis. J. Infect. Dis..

[14] McKenzie R, Maarsingh JD, Laniewski P, Herbst-Kralovetz MM (2021). Immunometabolic analysis of *Mobiluncus mulieris* and *Eggerthella* sp. Reveals novel insights into their pathogenic contributions to the hallmarks of bacterial vaginosis. Front. Cell Infect. Microbiol..

[15] Spiegel CA, Davick P, Totten PA, Chen KC, Eschenbach DA, Amsel R, Holmes KK (1983). Gardnerella vaginalis and anaerobic bacteria in the etiology of bacterial (nonspecific) vaginosis. Scand. J. Infect. Dis. Suppl..

[16] Hardy L, Jespers V, Van den Bulck M, Buyze J, Mwambarangwe L, Musengamana V, Vaneechoutte M, Crucitti T (2017). The presence of the putative *Gardnerella vaginalis* sialidase A gene in vaginal specimens is associated with bacterial vaginosis biofilm. PLoS ONE.

[17] van de Wijgert JH, Borgdorff H, Verhelst R, Crucitti T, Francis S, Verstraelen H, Jespers V (2014). The vaginal microbiota: What have we learned after a decade of molecular characterization?. PLoS ONE.

[18] Fredricks DN, Fiedler TL, Thomas KK, Oakley BB, Marrazzo JM (2007). Targeted PCR for detection of vaginal bacteria associated with bacterial vaginosis. J. Clin. Microbiol..

[19] Castro J, Jefferson KK, Cerca N (2020). Genetic heterogeneity and taxonomic diversity among gardnerella species. Trends Microbiol..

[20] Vaneechoutte M, Guschin A, Van Simaey L, Gansemans Y, Van Nieuwerburgh F, Cools P (2019). Emended description of *Gardnerella vaginalis* and description of *Gardnerella leopoldii* sp. nov., *Gardnerella piotii* sp. nov. and *Gardnerella swidsinskii* sp. nov., with delineation of 13 genomic species within the genus Gardnerella. Int. J. Syst. Evol. Microbiol..

[21] Plummer EL, Vodstrcil LA, Murray GL, Fairley CK, Danielewski JA, Garland SM, Chow EPF, Bulach DM, Fethers KA, Hocking JS (2020). *Gardnerella vaginalis* clade distribution is associated with behavioral practices and nugent score in women who have sex with women. J. Infect. Dis..

[22] Qin H, Xiao B (2022). Research progress on the correlation between gardnerella typing and bacterial vaginosis. Front. Cell Infect. Microbiol..

[23] Hill JE, Albert AYK, Group VR (2019). Resolution and cooccurrence patterns of *Gardnerella leopoldii*, *G. swidsinskii*, *G. piotii*, and *G. vaginalis* within the vaginal microbiome. Infect. Immun..

[24] Machado D, Castro J, Palmeira-de-Oliveira A, Martinez-de-Oliveira J, Cerca N (2015). Bacterial vaginosis biofilms: Challenges to current therapies and emerging solutions. Front. Microbiol..

[25] Rosca AS, Castro J, Sousa LGV, Franca A, Cavaleiro C, Salgueiro L, Cerca N (2022). Six bacterial vaginosis-associated species can form an in vitro and ex vivo polymicrobial biofilm that is susceptible to *Thymbra capitata* essential oil. Front. Cell Infect. Microbiol..

[26] Bradshaw CS, Sobel JD (2016). Current treatment of bacterial vaginosis-limitations and need for innovation. J. Infect. Dis..

[27] Zimmermann P, Curtis N (2019). The effect of antibiotics on the composition of the intestinal microbiota—a systematic review. J. Infect..

[28] Faught BM, Reyes S (2019). Characterization and treatment of recurrent bacterial vaginosis. J. Womens Health (Larchmt).

[29] Wu S, Hugerth LW, Schuppe-Koistinen I, Du J (2022). The right bug in the right place: Opportunities for bacterial vaginosis treatment. NPJ Biofilms Microbiomes.

[30] Dover SE, Aroutcheva AA, Faro S, Chikindas ML (2008). Natural antimicrobials and their role in vaginal health: A short review. Int. J. Probiot. Prebiot..

[31] Sousa LGV, Castro J, Cavaleiro C, Salgueiro L, Tomas M, Palmeira-Oliveira R, Martinez-Oliveira J, Cerca N (2022). Synergistic effects of carvacrol, alpha-terpinene, gamma-terpinene, rho-cymene and linalool against Gardnerella species. Sci. Rep..

[32] Machado D, Gaspar C, Palmeira-de-Oliveira A, Cavaleiro C, Salgueiro L, Martinez-de-Oliveira J, Cerca N (2017). Thymbra capitata essential oil as potential therapeutic agent against *Gardnerella vaginalis* biofilm-related infections. Future Microbiol..

[33] Mathai JK, Liu Y, Stein HH (2017). Values for digestible indispensable amino acid scores (DIAAS) for some dairy and plant proteins may better describe protein quality than values calculated using the concept for protein digestibility-corrected amino acid scores (PDCAAS). Br. J. Nutr..

[34] Esposito E, Campolo M, Casili G, Lanza M, Filippone A, Peritore AF, Cuzzocrea S (2018). Effect of pea protein plus grape seed dry extract on a murine model of *Candida albicans* induced vaginitis. Future Microbiol..

[35] Gilbert NM, Lewis WG, Lewis AL (2013). Clinical features of bacterial vaginosis in a murine model of vaginal infection with *Gardnerella vaginalis*. PLoS ONE.

[36] Carvalho FC, Bruschi ML, Evangelista RC, Gremiao MPD (2010). Mucoadhesive drug delivery systems. Braz. J. Pharm. Sci..

[37] Bagnall P, Rizzolo D (2017). Bacterial vaginosis: A practical review. JAAPA.

[38] Medina-Colorado AA, Vincent KL, Miller AL, Maxwell CA, Dawson LN, Olive T, Kozlova EV, Baum MM, Pyles RB (2017). Vaginal ecosystem modeling of growth patterns of anaerobic bacteria in microaerophilic conditions. Anaerobe.

[39] Ma B, Forney LJ, Ravel J (2012). Vaginal microbiome: Rethinking health and disease. Annu. Rev. Microbiol..

[40] Doyle R, Gondwe A, Fan YM, Maleta K, Ashorn P, Klein N, Harris K (2018). A lactobacillus-deficient vaginal microbiota dominates postpartum women in rural Malawi. Appl. Environ. Microbiol..

[41] Plesniarski A, Siddik AB, Su RC (2021). The microbiome as a key regulator of female genital tract barrier function. Front. Cell Infect. Microbiol..

[42] Yarbrough VL, Winkle S, Herbst-Kralovetz MM (2015). Antimicrobial peptides in the female reproductive tract: A critical component of the mucosal immune barrier with physiological and clinical implications. Hum. Reprod. Update.

[43] Tomas M, Palmeira-de-Oliveira A, Simoes S, Martinez-de-Oliveira J, Palmeira-de-Oliveira R (2020). Bacterial vaginosis: Standard treatments and alternative strategies. Int. J. Pharm..

[44] Oduyebo OO, Anorlu RI, Ogunsola FT (2009). The effects of antimicrobial therapy on bacterial vaginosis in non-pregnant women. Cochrane Database Syst. Rev..

[45] Reiter S, Kellogg Spadt S (2019). Bacterial vaginosis: A primer for clinicians. Postgrad. Med..

[46] Stan D, Enciu AM, Mateescu AL, Ion AC, Brezeanu AC, Stan D, Tanase C (2021). Natural compounds with antimicrobial and antiviral effect and nanocarriers used for their transportation. Front. Pharmacol..

[47] Bulgasem BY, Lani MN, Hassan Z, Wan Yusoff WM, Fnaish SG (2016). Antifungal activity of lactic acid bacteria strains isolated from natural honey against pathogenic Candida species. Mycobiology.

[48] Boukhatem MN, Setzer WN (2020). Aromatic herbs, medicinal plant-derived essential oils, and phytochemical extracts as potential therapies for coronaviruses: Future perspectives. Plants (Basel).

[49] Rosenfeld WD, Clark J (1989). Vulvovaginitis and cervicitis. Pediatr. Clin. N. Am..

[50] Barnhart KT, Pretorius ES, Timbers K, Shera D, Shabbout M, Malamud D (2004). In vivo distribution of a vaginal gel: MRI evaluation of the effects of gel volume, time and simulated intercourse. Contraception.

[51] Paterniti I, Casili G, Filippone A, Lanza M, Ardizzone A, Capra AP, Campolo M, Esposito E (2022). A new approach for the treatment of recurrent vulvovaginal candidiasis with a combination of pea protein, grape seed extract, and lactic acid assessed in vivo. J. Fungi (Basel).

[52] Briselden AM, Moncla BJ, Stevens CE, Hillier SL (1992). Sialidases (neuraminidases) in bacterial vaginosis and bacterial vaginosis-associated microflora. J. Clin. Microbiol..

[53] Lewis AL, Lewis WG (2012). Host sialoglycans and bacterial sialidases: A mucosal perspective. Cell Microbiol..

[54] Bautista CT, Wurapa EK, Sateren WB, Morris SM, Hollingsworth BP, Sanchez JL (2017). Association of bacterial vaginosis with chlamydia and gonorrhea among women in the US army. Am. J. Prev. Med..

[55] Kim H, Kim Y, Kang CH (2021). In vivo confirmation of the antimicrobial effect of probiotic candidates against *Gardnerella vaginalis*. Microorganisms.

[56] Jang SE, Jeong JJ, Choi SY, Kim H, Han MJ, Kim DH (2017). *Lactobacillus rhamnosus* HN001 and *Lactobacillus acidophilus* La-14 attenuate *Gardnerella vaginalis*-infected bacterial vaginosis in mice. Nutrients.

[57] Valentim AM, Guedes SR, Pereira AM, Antunes LM (2016). Euthanasia using gaseous agents in laboratory rodents. Lab Anim.

[58] Choi SI, Won G, Kim Y, Kang CH, Kim GH (2022). Lactobacilli strain mixture alleviates bacterial vaginosis through antibacterial and antagonistic activity in gardnerella vaginalis-infected C57BL/6 mice. Microorganisms.

[59] Anton L, Ferguson B, Friedman ES, Gerson KD, Brown AG, Elovitz MA (2022). Gardnerella vaginalis alters cervicovaginal epithelial cell function through microbe-specific immune responses. Microbiome.

[60] Lanza M, Casili G, Torre GL, Giuffrida D, Rotondo A, Esposito E, Ardizzone A, Rando R, Bartolomeo G, Albergamo A (2020). Properties of a new food supplement containing actinia equina extract. Antioxidants (Basel).

[61] O'Brien VP, Gilbert NM, Lebratti T, Agarwal K, Foster L, Shin H, Lewis AL (2019). Low-dose inoculation of *Escherichia coli* achieves robust vaginal colonization and results in ascending infection accompanied by severe uterine inflammation in mice. PLoS ONE.

[62] Zhang JE, Luo D, Chen RY, Yang YP, Zhou Y, Fan YM (2013). Feasibility of histological scoring and colony count for evaluating infective severity in mouse vaginal candidiasis. Exp. Anim..

[63] Wu S, Lin X, Hui KM, Yang S, Wu X, Tan Y, Li M, Qin AQ, Wang Q, Zhao Q (2019). A biochemiluminescent sialidase assay for diagnosis of bacterial vaginosis. Sci. Rep..

[64] Robinson LS, Schwebke J, Lewis WG, Lewis AL (2019). Identification and characterization of NanH2 and NanH3, enzymes responsible for sialidase activity in the vaginal bacterium *Gardnerella vaginalis*. J. Biol. Chem..

